# The foot-health of people with diabetes in regional and rural Australia: baseline results from an observational cohort study

**DOI:** 10.1186/s13047-019-0366-6

**Published:** 2019-12-05

**Authors:** Byron M. Perrin, Penny Allen, Marcus J. Gardner, Andrew Chappell, Bronwyn Phillips, Claire Massey, Isabelle Skinner, Timothy C. Skinner

**Affiliations:** 1 La Trobe Rural Health School, La Trobe University, PO Box 199, Bendigo, 3552 Australia; 2 Rural Clinical School, University of Tasmania, Burnie, Australia; 3 Clinical Learning and Development, Bendigo Health, Bendigo, Australia; 4 Tasmanian Health Service- North West, Burnie, Australia; 5 Murray Primary Health Network, Bendigo, Australia; 6 Tasmanian Health Service- North, Launceston, Australia; 7 Centre for Rural and Remote Health, James Cook University, Townsville, Australia; 8 Department of Psychology, University of Copenhagen, Copenhagen, Denmark

**Keywords:** Podiatry, Diabetes, Models of care, Diabetic foot, Australia

## Abstract

**Background:**

There is limited Australian epidemiological research that reports on the foot-health characteristics of people with diabetes, especially within rural and regional settings. The objective of this study was to explore the associations between demographic, socio-economic and diabetes-related variables with diabetes-related foot morbidity in people residing in regional and rural Australia.

**Methods:**

Adults with diabetes were recruited from non-metropolitan Australian publicly-funded podiatry services. The primary variable of interest was the University of Texas diabetic foot risk classification designated to each participant at baseline. Independent risk factors for diabetes-related foot morbidity were identified using multivariable analysis.

**Results:**

Eight-hundred and ninety-nine participants enrolled, 443 (49.3%) in Tasmania and 456 (50.7%) in Victoria. Mean age was 67 years (SD 12.7), 9.2% had type 1 diabetes, 506 (56.3%) were male, 498 (55.4%) had diabetes for longer than 10 years and 550 (61.2%) either did not know the ideal HbA1c target or reported that it was ≥7.0. A majority had peripheral neuropathy or worse foot morbidity (61.0%). Foot morbidity was associated with male sex (OR 2.42, 95% CI 1.82–3.22), duration of diabetes > 20 years (OR 3.25, 95% CI 2.22–4.75), and Tasmanian residence (OR 3.38, 95% CI 2.35–4.86).

**Conclusions:**

A high proportion of the regional Australian clinical population with diabetes seen by the publicly-funded podiatric services in this study were at high risk of future limb threatening foot morbidity, and participants residing in Northern Tasmania are more likely to have worse diabetes-related foot morbidity than those from regional Victoria. Service models should be reviewed to ensure that diabetes-related foot services are appropriately developed and resourced to deliver interdisciplinary evidence-based care.

## Background

People with diabetes mellitus can develop complications to the lower limb and feet such as peripheral neuropathy, [1] peripheral arterial disease, [2] Charcot neuropathic osteoarthropathy, [3] foot ulceration, [4] and lower limb amputation [5]. Diabetes-related foot complications rank second behind kidney disease in the burden of disease for diabetes complications in Australia, [6] and costs an estimated A$1.6 billion each year [7]. This equates to an estimated 1.1% of the Australian national health budget, [8] a proportion similar to that estimated in the United Kingdom [9]. As a cause of hospital admission, people with foot complications require inpatient length of stays longer than any other diabetes complication [10].

Population-based data from the AusDiab study suggest the prevalence of peripheral neuropathy in Australia to be 13% in people with known diabetes, [11] with an increased prevalence of 21% in specialist diabetes clinical populations [12]. It is estimated that 1.9–5.3% of people with diabetes in Australia will have experienced a diabetes-related foot ulcer [13]. Diabetes-related foot ulceration resulted in nearly 10,000 Australian hospital admissions in 2005, with 8% of all diabetes-related deaths in that year being a direct result of diabetes-related ulceration [14]. More recently, diabetic foot disease (ulceration, ischaemia, infection and Charcot neuropathic osteoarthropathy) is estimated to result in 27,600 hospital admissions each year [15]. The number of diabetes-related lower-limb amputations performed in Australia has increased from approximately 2600 each year for the years 1995–1998 [16] to a 2017 estimate of 4400 [15].

Diabetes-related lower limb complications are disproportionally found in socially disadvantaged and Indigenous populations. A retrospective Victorian study found a significantly higher rate of hospital admissions for people with diabetes from geographical areas of greater social disadvantage, [17] and Australian Institute of Health and Welfare statistics suggest a 50% higher probability of lower limb amputation for people residing in rural areas [18].

To date, there is limited prospective epidemiological research that reports on the foot-health characteristics of people with diabetes, especially within rural and regional settings. The objective of this analysis was to explore the associations between demographic, socio-economic and diabetes-related variables with diabetes-related foot morbidity in people residing in regional and rural Australia.

## Methods

### Design

This study was approved by the Human Research Ethics Committees of the University of Tasmania, Bendigo Health and La Trobe University. This article reports the baseline characteristics of those recruited to a longitudinal, prospective cohort study- *a prospective cohort study of diabetes-related foot-health in Australian rural areas* (*PoDFAR*). The cohort of participants were conveniently recruited from publicly-funded podiatric services in regional and rural Victoria and Tasmania over two time periods of three-months during 2013 (March–May and September–November). For the podiatry services involved in the study, all patients aged over 18 years with diabetes who presented during the recruitment periods were consecutively invited to participate, with written informed consent gained prior to enrolment.

### Setting

The podiatric services involved in the study are found within the Greater Bendigo area of the Loddon Mallee region of central Victoria, and the North and North-West areas of Tasmania. It is estimated that 7.5% of the Loddon Mallee population and 8.1% of the Northern Tasmanian population have a diagnosis of diabetes, which is higher than the Australian estimate of 4.9% [19–21]. Data collection was undertaken by multiple podiatry services of four publicly funded health organisations. At the time of the study there were 9.5 full-time-equivalent podiatrists employed in central Victoria and 8.3 in northern Tasmania, with the health services being delivered across a regional catchment area of 300,000 people in Victoria [22] and 255,000 people in Tasmania [23]. Service delivery operations range from large regional hospitals to community health centres, primary health care settings and outreach programs.

Podiatry services are funded through a mix of state and federal government sources, and this can influence eligibility and the profile of patients seen in each service. In general, demand for services outstrips supply and priority referral policies are also influential. The services can be described as community-based (including home visits), subacute or hospital inpatient care. There is a similar staff profile working in each of these settings across the two states. In both states, community services take up a large proportion of the employed podiatry staff (82.1% in Victoria and 69.9% in Tasmania). Although the proportion of staff employed is low in subacute settings in both states (13.7% in Victoria and 18.1% in Tasmania), previous Victorian research involving the same podiatric services suggests that these services deal with patients with more severe diabetes-related foot morbidity, with more occasions of service per patient than the other settings [24].

In Victoria, the included podiatric services formally approach the care of people with diabetes using an established Podiatry Diabetes Model (PDM) [25]. The PDM is a health model of care developed with the aim of assisting efficient and effective management of patients with diabetes across the entire foot-health risk spectrum. The model has a set of associated local clinical guidelines adapted from national guidelines [26] to provide robust evidence-based clinical parameters for podiatrist clinical decision making. The PDM is underpinned by all podiatrists classifying a patient’s foot-health at each point of contact using the validated University of Texas (UT) diabetic foot risk classification tool (Table 1) [27, 28]. This classification tool facilitates clinical decision making that considers key risk factors for ulceration (neuropathy, foot deformity and prior history of limb threatening diabetes-related foot pathology) and lower limb amputation (current ulceration, Charcot neuro-osteoarthropathy, infection and ischaemia). The use of this tool also facilitates the movement of a patient to the most appropriate podiatric service by way of the validated PDM referral pathway [24].

**Table 1:** The University of Texas Risk Classification [27, 28]

0 No neuropathy	1 Peripheral neuropathy	2 Neuropathy with deformity	3 History of pathology
4A Foot ulceration	4B Acute Charcot arthropathy	5 Infected foot	6 Ischaemia

In Tasmania, the podiatric services use the UT diabetic foot risk classification tool in accordance with the clinical underpinnings of the PDM. The referral pathway of the PDM was not adopted during the study and the clinical guidelines are used as a guide only. The use of the UT diabetic foot risk classification by the Tasmanian podiatrists commenced 9 months prior to recruitment. Transition to the new tool was facilitated by two days of formal training by the developers of the PDM.

### Data collection

At the time of the first recruitment period all participating podiatric services were classifying the foot health of their patients using the UT risk tool as standard practice at every point of contact. For the enrolled participants, this foot-health information and other demographic, diabetes-related and socio-economic variables were recorded at baseline. Data collection involved the adoption of point-of-contact data collection through the use of tablet devices electronically linked to a central database. Each podiatrist in each podiatry service had access to a tablet device to record the variables of interest in a survey application. At the end of each week all new data collected by podiatrists were uploaded to a central database for collation.

The primary variable of interest was the UT diabetic foot risk classification designated to each enrolled participant at their baseline visit. The UT risk classification system was chosen as it has been shown to be a reliable, valid and predictive tool for identifying future foot-health outcomes for people with diabetes [27, 28]. Demographic and diabetes-related variables of interest were age, sex, state of residence, diabetes type and duration, participants’ self-reported knowledge of optimal blood glucose control and current smoking status. Postcode was used to classify participants’ residence according to Australian Bureau of Statistics (ABS) Australian Statistical Geography Standard (ASGS) [29].

Postcodes were also matched to ABS Socio-Economic Indexes for Areas, Index of Relative Socio-Economic Disadvantage (IRSD) deciles to derive an estimation of level of social disadvantage [30]. IRSD takes into account the collective characteristics of an area’s resident group, including, among other variables, employment status, housing expenditure, disability and one-parent households. IRSD scores (highest proportion of disadvantage) are ranked in deciles, with lower deciles indicating higher levels of social disadvantage.

### Statistical analysis

Data collected was centrally collated using the survey application. This data was exported into an Excel spreadsheet, checked for accuracy and cleaned. The data was then imported into Stata 15 (StataCorp: College Station, Texas) for analysis. Data distributions were investigated and summarised using means and standard deviations (SD) for parametric continuous data, medians and interquartile ranges (IQR) for non-parametric data and frequencies with percentages for categorical data. The number of UT risk categories was consolidated into three groups: “no neuropathy” (UT category 0), “neuropathy, no prior history of pathology” or “neuropathy with deformity” (UT categories 1 and 2) and “history of, or current pathology” (UT categories 3–6). The consolidation of risk categories was undertaken to allow for more robust statistical comparison of patient risk profiles in line with evidence-based risk for future ulceration or lower limb amputation- the primary outcome measures for the follow-up component of the cohort study.

Bivariate analyses were undertaken to investigate demographic, diabetes-related and socio-economic variables within the Victorian and Tasmanian samples, and variables associated with consolidated UT risk category. Categorical variables were investigated using Chi-square tests. Non-parametric age data was square transformed prior to running a one-way analysis of variance (ANOVA) for risk group comparisons and the independent *t-*test was used for state-based comparisons. The Kruskal-Wallis test was used to investigate differences in median IRSD deciles between the three consolidated risk groups. Variables that were significant on bivariate analyses were entered into an ordered logistic regression and the parallel regression assumption was investigated using the Brant test. This indicated age (squared) and state of residence violated the parallel assumption. A generalised ordered logit model was subsequently produced and adjusted odds ratios with 95% confidence intervals were generated. All tests were two-sided and differences were accepted at *p* < 0.05 significance level.

A study sample size power calculation was conducted during the design phase of the cohort study. This paper presents descriptive analyses of baseline characteristics, as part of the larger cohort study. Using a conservative rule-of-thumb of 50 events per variable, with 7 independent variables plus *n* = 100 to calculate the minimum sample size for logistic regression (*n* = 450), adjusting this for an ordinal logistic regression the minimal sample size for our model is 675. As 899 participants were included in the model, we are confident that the model had sufficient power.

## Results

The study recruited 899 patients with diabetes mellitus (Table 2). There were 506 (56.3%) male participants and mean age was 67 years (SD 12.7). Fifty-five percent of participants had been diagnosed with diabetes for longer than 10 years and 423 (47.1%) did not know an optimal HbA1c target. Ranked levels of disadvantage show a median IRSD decile rank of 3 (range 1–9) with 58.7% of participants residing in the third most deprived postcodes (IRSD deciles 1–3). The proportion of participants recruited was equivalent in each state. There was a spread of participants across all the individual UT Texas risk categories, with the majority of the sample having neuropathy or a worse pathology (*n* = 548, 61.0%).

**Table 2:** Participant characteristics of *n* = 899

Variable	Mean (SD) or n (%) or Median (IQR)
Age (years)	67.0 (12.7)
Sex (%)	
Male	506 (56.3)
Female	393 (43.7)
Diabetes type (%)	
Type 1	83 (9.2)
Type 2	816 (90.8)
Diabetes duration (years)	
Newly diagnosed	38 (4.2)
1–5	183 (20.4)
6–10	165 (18.4)
11–15	177 (19.7)
16–20	99 (11.0)
> 20 years	222 (24.7)
Unknown	15 (1.7)
Current smoker	
Yes	114 (12.7)
No	785 (87.3)
Self-reported ideal HbA1c target (mmol/L)	
< 7.0	349 (38.8)
7.0–7.5	59 (6.6)
7.6–8.0	28 (3.1)
> 8.0	40 (4.4)
Don’t know	423 (47.1)
IRSD decile (median (IQR))	3 (2–4)
Rurality of residence (ASGC)	
Metropolitan (RA1)	0 (−)
Inner regional (RA2)	571 (63.5)
Outer regional (RA3)	293 (32.6)
Remote (RA4)	26 (2.9)
Very remote (RA5)	9 (1.0)
State of residence	
Tasmania	443 (49.3)
Victoria	456 (50.7)
UT Risk Classification	
No neuropathy	351 (39.0)
Neuropathy	149 (16.6)
Neuropathy and deformity	85 (9.5)
History of pathology	122 (13.6)
Neuropathic ulcer	104 (11.6)
Acute Charcot	8 (0.9)
Infection	25 (2.8)
Ischaemia	55 (6.1)

After risk category consolidation, there were 351 (39.0%) participants with no neuropathy at baseline, 234 participants (26.0%) with neuropathy and no prior history of pathology and 314 (34.9%) with history of or current pathology (Table 3). Bivariate analyses found women comprised 57.3% of the group with no neuropathy, while 70.7% of those with a history of pathology or current pathology were men (χ^2^ (2) = 52.8, *p* < 0.0001). There was no significant difference in the proportion of smokers in each risk group (χ^2^ (2) = 4.5, *p* = 0.10). There was also no difference in median IRSD decile between the groups (*H* = 2.2, *p* = 0.33). All other variables were associated with UT risk group.

**Table 3:** Bivariate relationship analysis of participant characteristics with UT score

Variable	No Neuropathy (*n* = 351)	Neuropathy/no prior history of pathology or neuropathy with deformity (*n* = 234)	History of or current pathology (*n* = 314)	*p*
Sex				
Male	150 (29.6)	134 (26.5)	222 (43.9)	< 0.0001^a^
Female	201 (51.1)	100 (25.4)	92 (23.4)	
Age	65 (13.5)	72 (10.7)	66 (12.0)	< 0.0001^a^
Diabetes type				
Type 1	26 (31.3)	12 (14.5)	45 (54.2)	< 0.0001^a^
Type 2	325 (39.8)	222 (27.2)	269 (33.0)	
Diagnosed > 20 years^+^	45 (20.3)	52 (23.4)	125 (56.3)	< 0.0001^a^
Current smoker	50 (44.6)	20 (17.9)	42 (37.5)	0.10
Knowledge of ideal HbA1c target (mmol/L)				
≤ 7.5	169 (41.4)	89 (21.8)	150 (36.8)	0.03^a^
> 7.5 or don’t know	182 (37.1)	145 (29.5)	164 (33.4)	
IRSD decile (median (IQR))	3 (2–4)	3 (2–4)	3 (2–5)	0.33
Rurality of residence				
Inner regional	223 (39.1)	166 (29.1)	182 (31.9)	0.01^a^
Outer regional	111 (37.9)	58 (19.8)	124 (42.3)	
Remote or very remote	17 (48.6)	10 (28.6)	8 (22.9)	
State of Residence				
Tasmania	159 (35.9)	74 (16.7)	210 (47.4)	< 0.0001^a^
Victoria	192 (42.1)	160 (35.1)	104 (22.8)	

Notes: Data presented as n (%) or mean (SD) or IRSD decile (median (IQR)). Percentages are reported by row. ^+^15 participants who could not provide duration of diabetes information excluded from this calculation. ^a^Statistically significant

The Victorian sample were over-represented in the neuropathy/neuropathy with deformity group (*n* = 160, 68.4%) while the Tasmanian sample comprised a larger proportion of those with a history of or current pathology (*n* = 210, 47.4%) (χ^2^ (2) = 70.3, *p* < 0.0001) (Table 4). Tasmanian participants were younger (*t*_(881)_ = − 6.9, *p* < 0.0001), more likely to be diagnosed with Type 1 diabetes (χ^2^ (1) = 21.5 *p* < 0.0001), living with diabetes for longer than 20 years (χ^2^ (1) = 9.2, *p* = 0.002) and residing in outer regional or remote areas (ASGS RA3-RA5) (χ^2^ (2) = 250.9, *p* < 0.0001). Victorian participants had poorer knowledge of ideal HbA1c level (χ^2^ (1) = 9.46, *p* = 0.002). This difference remained after excluding those with Type 1 diabetes, with 61.3% of Victorian participants with type 2 diabetes reporting an incorrect or unknown ideal target HbA1c level, compared to 52.9% of Tasmanian participants with type 2 diabetes (χ^2^ (1) = 5.88, *p* = 0.02).

**Table 4:** State-based participant foot-health characteristics

Variable	Tasmania (*n* = 443)	Victoria (*n* = 456)	*p*
Sex			
Male	262 (59.1)	244 (53.5)	0.09
Female	181 (40.9)	212 (46.5)	
Age	64 (12.9)	70 (11.7)	< 0.0001^a^
Diabetes type			
Type 1	61 (13.8)	22 (4.8)	< 0.0001^a^
Type 2	382 (86.2)	434 (95.2)	
Diagnosed > 20 years^+^	129 (29.6)	93 (20.8)	0.002^a^
Current smoker	51 (11.5)	61 (13.4)	0.40
Knowledge of ideal HbA1c target (mmol/L)			
≤ 7.5	224 (50.6)	184 (40.4)	0.002^a^
> 7.5 or don’t know	219 (49.4)	272 (55.4)	
IRSD decile (median (IQR))	3 (2–5)	3 (2–4)	0.33
Rurality of residence			
Inner regional	168 (37.9)	403 (88.4)	
Outer regional	240 (54.2)	53 (11.6)	< 0.0001^a^
Remote or very remote	35 (7.9)	0 (−)	
UT Risk Classification			
No neuropathy	159 (35.9)	192 (42.1)	N/A
Neuropathy	63 (14.2)	86 (18.9)	
Neuropathy with deformity	11 (2.5)	74 (16.2)	
History of pathology	79 (17.8)	43 (9.4)	
Neuropathic ulcer	67 (15.1)	37 (8.1)	
Acute Charcot	6 (1.4)	2 (0.4)	
Infection	19 (4.3)	6 (1.3)	
Ischaemia	39 (8.8)	16 (3.5)	
Consolidated UT Risk Classification			
No neuropathy	159 (35.9)	192 (42.1)	
Neuropathy/neuropathy with deformity	74 (16.7)	160 (35.1)	< 0.0001^a^
History of, or current, pathology	210 (47.4)	104 (22.8)	

Notes: Data presented as n (%) or mean (SD) or IRSD decile (median (IQR)). Percentages are reported by column. ^+^15 participants who could not provide duration of diabetes information excluded from this calculation. N/A: not reported due to small expected cell frequencies. ^a^Statistically significant

### Multivariable analysis of risk factors associated with consolidated UT risk group

Gender, age, diabetes type, duration of diagnosis (≤ 20 years vs. > 20 years), knowledge of optimal HbA1c level (< 7.5 vs. don’t know or ≥ 7.5), rurality and state of residence were entered into a generalised ordered logit model with consolidated UT risk group (no neuropathy, neuropathy and deformity or history of/current pathology) as the dependent variable. The overall model was significant [likelihood ratio χ^2^ (14) = 220.7, *p* < 0.0001, pseudo R^2^ = 0.12]. Diabetes type and knowledge of ideal HbA1c level were not significantly associated with UT risk group. The adjusted odds ratio (OR) for men being in the history of, or current, pathology group was 2.42 (95% CI 1.82–3.22), for those diagnosed with diabetes > 20 years OR 3.25 (95% CI 2.22–4.75), remote or very remote residence versus inner and outer regional residence OR 0.65 (95% CI 0.48–0.87) and Tasmanian residence (OR 3.38, 95% CI 2.35–4.86).

## Discussion

This is a large cross-sectional analysis of a regional Australian podiatric clinical population. The clinical services involved in the study are led by podiatrists, with some input from other health professionals such as doctors, nurses and other allied health professionals. As expected for this clinical population the podiatric services are dealing with a high proportion (35.1%) of people who have a current or prior limb threatening pathology, which is much higher than the population-based AusDiab study [11]. In all clinics involved a priority system was in place to facilitate access to the services according to most need, and in Victoria the referral pathways between the two organisations are clearly defined by the PDM. Understanding the risk profile of a service is important, because the predominate independent risk factor for future diabetes-related limb threatening pathology is a prior history of limb threatening pathology, with the validation of the UT Texas risk classification demonstrating a 36-fold increase in risk for ulceration for people with a history of ulceration [28]. It is these patients that require a multi-disciplinary approach to their foot care the most.

Unfortunately, the high prevalence of foot morbidity in this regional and rural clinical population is consistent with previous research from regional Victoria, which has some of the highest rates of hospital admissions related to diabetes-related foot pathology in the state of Victoria [17]. Other research conducted in 2010 of a sample of over one hundred people with diabetes in the region who attended a subacute service involved in the study showed an annual incidence of diabetes-related foot ulceration of over 30 %, another very high fig [31]. In general, our sample had high levels of social disadvantage. This is consistent with previous research in this area which has shown a higher incidence of diabetic foot-related hospital admissions in less advantaged areas of Victoria [17]. A recent review of global incidence rates of diabetes-related lower limb amputations has also found that social deprivation may be significant in the development of diabetes-related foot pathology [32].

The 12.7% of participants who reported being a current smoker in this sample was slightly lower than the population-based National Drug Strategy Household Survey [33]. Smoking rates in diabetes tend to parallel that of the general population, however smokers with diabetes are at a much higher risk of morbidity and premature death associated with macrovascular complications [34]. What was particularly disappointing was knowledge of diabetes as measured by the participants reporting what the ideal Hb1Ac level should be, with nearly half the participants not knowing at all what an ideal level of diabetes control should be. Asking people with diabetes about what they think their ideal Hb1Ac should be is a simple, easy question to ask in clinical practice to identify individuals who might benefit from attending a structured self-management education programs, such as the Dose Adjustment for Normal Eating or the DESMOND programs [35, 36]. The results from this study indicate that referral pathways for regional and rural populations should specifically include access to diabetes education programs.

Interestingly, in bivariate analysis, participants with current or prior diabetes-related foot pathology were shown to be significantly younger than those in the neuropathy/neuropathy with deformity group. A plausible explanation is that those with current or prior diabetes-related foot pathology had a significantly longer duration of diabetes than the other groups, indicating that a longer duration diabetes was more influential in their pathology development than the fact that they were younger. This is why after multivariable analysis, age failed to remain an independent predictor of poor foot health.

### Independent risk associations with worse foot morbidity

Males were at more than double the risk of females of being in the current or prior history of pathology group. This is consistent with the literature. Population-based cohort and case-control studies from Australia and around the world have found that males have higher levels of peripheral neuropathy, [37, 38] foot ulceration, [31, 39] infection [40] and lower limb amputation [41, 42]. Men tend to seek access to health services less than females, perceive that they have less time for their own health and will engage in fewer health-promoting activities [43]. Duration of diabetes is an important predictor of foot health in this study, with diabetes duration of over 20 years increasing the risk of having a current or prior pathology three-fold. This is consistent with the AusDiab study, [11] which showed longer duration of diabetes to be associated with higher risk, and the known natural history of diabetes-related foot pathology [38].

This sample had generally high levels of risk for diabetes-related foot pathology, consistent with other regional and rural Australian data [18]. Although bivariate analysis found that level of remoteness was associated with poorer foot health, after the multivariable analysis (controlling for sex and duration of disease), remoteness was associated with better foot health. This suggests that poorer foot-health is less associated with rurality, and more a function of the characteristics of the patients that are attending the podiatric clinics in the regional and rural geographical areas. This is consistent with the large Australian Diabetes-MILES study, where remoteness was not associated with worse reported health problems or self-care indicators [44].

Those residing in the state of Tasmania were at more than three times the risk of having a current or prior history of serious diabetes-related foot pathology. This may be explained by the Tasmanian podiatric services model, where a higher proportion of podiatrists are employed in a subacute or acute setting attending to patients at higher risk of foot morbidity, as opposed to the Victorian services.

### Implications

This study has shown that regional publicly funded podiatric services in Australia are managing large numbers of patients at high risk of diabetes-related foot complications. National guidelines suggest that patients at high risk of diabetes-related foot pathology should have involvement with dedicated multi-disciplinary teams [15, 26]. However, three-quarters of the services involved in this study utilise community-based funding for the employment of podiatrists. Multi-disciplinary care is rarely the focus of Australian community-based podiatric services, which are more based on single discipline primary care model. These findings suggest that a high proportion of patients with diabetes seen by the podiatric services in this study were at high risk of future limb threatening foot morbidity, funded by programs that may not allow support for patients according to best recommendations [15, 26].

Further expansion of multi-disciplinary clinics, particularly outside of metropolitan and larger regional centres, may not be achievable in the medium term. What could be achieved is improved utilisation of standardised clinical guidelines, particularly as Australian research has shown that in community podiatry settings clinical guidelines are under-utilised [45]. There are established and robust risk systems and guidelines that have been developed that can inform evidenced-based practice in regional and rural podiatry-led services, such as the UT risk classification tool used in his study, or more recently published guidelines from the International Working Group on the Diabetic Foot [46]. The adoption of the PDM is an example of health organisations in a local area implementing models of care that use clinical guidelines and risk stratification tools to guide patient access to appropriate services and standardised care. The PDM provides a relatively simple conceptual example for the provision of podiatric services across different health system levels for people with diabetes in regional Australia [25]. The use of the risk classification system ensures the model has a sound evidence base, and the use of the available health service resources are driven by the clinical needs of the patients matched with the appropriate skill-set of the service providers. The podiatric care of people with diabetes in Australia requires the development of a seamless and collaborative model of care established within and across different health settings, with a clear definition of what role each health service has with respect to the obligations to the patient group [7, 45].

### Limitations

This study included a population with diabetes who attended predominately outpatient podiatric clinical settings in regional and rural Australia. As such, care should be taken when generalising to other populations, such as metropolitan, hospital inpatients or the general population of people with diabetes in Australia. This is particularly pertinent when considering people with diabetes in the community who have not developed peripheral neuropathy or peripheral arterial disease. This population is at a low risk of developing a limb threatening complication, and foot health care and prevention strategies do not require a dedicated multi-disciplinary service. The International Working Group on the Diabetic Foot provides strong evidence to recommend annual screening for loss of protective sensation and peripheral arterial disease, with education to be provided about appropriate foot self-care and identification of pre-ulceration signs [47].

Another limitation to this study is the lack of objective measurement of blood glucose control. Unfortunately, it was not practical to objectively measure blood glucose control in this study. The podiatry clinics do not consistently measure blood glucose control as this is primarily the responsibility of the primary health care giver (general practitioners). Causal links between poor glucose control and the development of peripheral neuropathy have been previously established [1] so it is probable that glucose control was correlated with levels of foot health in this study. However, this was unable to be quantified, so it is possible that glucose control could be a confounding variable in the multivariate analysis. This might particularly be the case for the strong association found between state of residence and foot health, where Tasmanian residents were over three times as likely to have had history of serious pathology. Further studies of this nature should include objective measurement of blood glucose.

## Conclusions

A high proportion of the regional Australian clinical population with diabetes seen by the publicly-funded podiatric services in this study were at high risk of future limb threatening foot morbidity, with participants residing in Northern Tasmania more likely to have worse diabetes-related foot morbidity than those from regional Victoria. Male sex and those diagnosed with diabetes > 20 years were at greater risk of diabetes-related foot morbidity. There was also a lack of knowledge demonstrated by the sample of basic diabetes information, indicating low levels of engagement in diabetes care and or little provision of effective high quality educational services to people with diabetes in these regional and rural areas.

The predominately community-funded podiatry services models underpinning these services are not well supported to provide the necessary multi-disciplinary care that the current evidence-base suggests is needed. This includes appropriate access to specialist medical and diabetes education in addition to the podiatry-lead services. Service models should be reviewed to ensure that diabetes-related foot services are appropriately developed and resourced to deliver sustainable multidisciplinary evidence-based care.

## Data Availability

The datasets used and/or analysed during the current study are available from the corresponding author on reasonable request.

## Abbreviations

ABS: Australian Bureau of Statistics
ANOVA: One-way analysis of variance
ASGS: Australian Statistical Geography Standard
IRSD: Index of Relative Socio-Economic Disadvantage
PDM: Podiatry Diabetes Model
PoDFAR: “a prospective cohort study of diabetes-related foot-health in Australian rural areas”
UT: University of Texas
